# Are volatile anesthetics neuroprotective or neurotoxic?

**DOI:** 10.1186/2045-9912-2-10

**Published:** 2012-04-17

**Authors:** Zhiyi Zuo

**Affiliations:** 1 Department of Anesthesiology, University of Virginia, 1 Hospital Drive, PO Box 800710, Charlottesville, VA 22908-0710, USA

**Keywords:** Anesthesia mechanism, Neuroprotection, Neurotoxicity, Preconditioning, Postconditioning, Volatile anesthetics

## Abstract

Volatile anesthetics are one class of the most commonly used drugs. However, the mechanisms for these drugs to induce anesthesia are not fully understood and have been under intensive investigation. Two other effects of these anesthetics on the central nervous system, volatile anesthetics-induced neuroprotection and neurotoxicity, currently are hot research fields. Although data from animal studies for these two effects are extensive and convincing, clinical data for volatile anesthetics-induced neuroprotection are relatively weak. There is essentially lack of evidence to suggest volatile anesthetics-induced neurotoxicity in humans. In this regard, the contribution of general anesthesia/anesthetics to postoperative cognitive decline, a clinical entity whose existence has been supported by substantial evidence, also has not been established. This paper will be focused on reviewing the evidence, especially the clinical evidence, for volatile anesthetics-induced neuroprotection and neurotoxicity. Efforts will be devoted to facilitating the understanding of the two seemingly contradictory effects of these important drugs on the brain.

## Introduction

More than 20 millions of patients each year have surgeries in the USA. The majority of these surgeries are performed under general anesthesia. About 80% of them receive volatile anesthetics as their primary anesthetics [1]. Since the first use of ether, a volatile anesthetic, in 1842, volatile anesthetics have be the major class of general anesthetics used in the clinical practice for near 160 years.

Although it is still controversial among the experts, it is generally accepted that general anesthesia minimally includes the following components: unconsciousness, insensateness, analgesia and amnesia. Many experts will also add muscle relaxation and bluntness of cardiovascular response to surgical stimulation into the components of general anesthesia. Volatile anesthetics, unlike most intravenous anesthetics, have pharmacological properties to provide all components of general anesthesia [2]. Thus, volatile anesthetics are full general anesthetics and, theoretically, single volatile anesthetic can be used to provide a patient with full general anesthesia for surgery. In addition, volatile anesthetics take effects very quickly. Most patients anesthetized by these drugs recover smoothly and quickly. With the aid of modern equipment, their use is very easy and their concentrations can be accurately monitored. For these reasons, volatile anesthetics have been popular drugs used in clinical practice. Modern volatile anesthetics that are used in the USA include isoflurane (CHF_2_-O-CHCl-CF_3_), sevoflurane (CH_2_F-O-CH-(CF_3_)_2_) and desflurane (CHF_2_-O-CHF-CF_3_). Halothane (CF_3_-CHBrCl) was used clinically for more than 40 years and started to be phased out during 1990s as newer volatile anesthetics become popular. All of these volatile anesthetics are halogenated hydrocarbons.

In addition to the anesthetic properties, volatile anesthetics have been thought to have neuroprotective effects for a long time [3,4]. Although the potential for volatile anesthetics to induce cell injury has been reported previously, there is a recent surge of concern on the safety of volatile anesthetics [5-7]. These two possible effects of volatile anesthetics, neuroprotection and neurotoxicity, and their implications in clinical practice will be discussed here. A brief overview of mechanisms of general anesthesia will be provided because providing general anesthesia is the main indication for using volatile anesthetics in humans and animals and this overview will facilitate the discussion of volatile anesthetics-induced neuroprotection and neurotoxicity.

## Overview of mechanisms for volatile anesthetics-induced anesthesia

Although general anesthetics are among the most commonly used drugs in clinical practice, the mechanisms for them to induce anesthesia are not fully understood. An early theory to explain anesthesia mechanism for volatile anesthetics is the Meyer-Overton rule [8]. It states that anesthetic potency increases with lipid solubility. This theory implies that general anesthetics are dissolved in the lipid fraction of brain cells to change the activity of these cells, which leads to anesthesia. Strong evidence to support the Meyer-Overton rule is the finding that there is a linear relationship between the solubility of volatile anesthetics in olive oil and their anesthetic potency [8]. Although the Meyer-Overton rule was the dominant theory to explain general anesthesia for many decades, many findings have questioned the correctness of this theory. For example, enantiomers of anesthetics have the same lipid solubility but different anesthetic potencies [9]. There are also many non-immobilizers that are similar to volatile anesthetics in chemical structures and lipid solubility but do not have significant anesthetic properties [10].

A very popular theory developed in the last 3 decades to explain general anesthesia is the protein hypothesis. It states that general anesthetics bind and act on specific proteins to change their functions and cell activity, which results in anesthesia [11]. Consistent with this hypothesis, functions/activities of numerous proteins have been found to be affected by anesthetics. These proteins include receptors, ion channels and neurotransmitter transporters whose changes in functions can alter the activity of the brain cells [2,11,12]. Since there are excitatory and inhibitory neurotransmissions in the central nervous system (CNS), a simply view of the protein hypothesis is that general anesthesia is induced by inhibiting the excitatory neurotransmission and/or enhancing the inhibitory neurotransmission. Since glutamate and γ-aminobutyric acid (GABA) are the major excitatory and inhibitor neurotransmitters, the role of their receptors in general anesthesia has been a focus of study in the last 3 decades [2,11,13].

The most convincing data obtained so far are on GABA receptors, especially the GABA_A _receptors. Multiple studies have shown that general anesthetics at clinically relevant concentrations enhance GABA receptor activity. Specific target sites in the receptors have been extensively studied by using site-directed mutagenesis [13,14]. It has been shown that S270 in the α2 subunit of the GABA_A _receptors is critically important for the enhancement of GABA_A _receptor activity by volatile anesthetics [15]. Mice with mutation on this amino acid residue have a reduced sensitivity to isoflurane [16]. Also, various studies with other mutations of the GABA_A _receptors in mice have suggested the importance of these receptors in anesthetic effects [14]. Finally, GABA_A _receptor antagonists have been shown to reverse anesthetic effects [17].

A few lines of evidence have shown the involvement of glutamate receptors, especially the N-methyl-D-aspartic acid (NMDA) receptors (a subtype glutamate receptors), in the mechanisms of general anesthesia. General anesthetics at clinically relevant concentrations can work as NMDA receptor antagonists [13,18]. Knockout of a subunit of the NMDA receptors in mice significantly reduces the anesthetic potency of nitrous oxide, an inhalation anesthetic [19]. Finally, blockage of NMDA receptors has been considered as the major mechanism for the effects of ketamine [20], an intravenous anesthetic.

Multiple other proteins, such as voltage-gated channels, background channels and neurotransmitter transporters, may be anesthetic targets [2,12,13,21]. This implication is mostly based on the evidence that anesthetics affect the activity of these proteins. In some cases, limited in vivo animal data are available to support their role in anesthesia mechanism. However, additional evidence is needed to establish this role for most of these proteins.

Associated with the protein hypothesis, identifying the mechanisms of general anesthesia at a system level has been a research focus in recent years. General anesthesia has been commonly described as "putting patients to sleep" in a layman term. In fact, sleep and general anesthesia share many features [22]. For example, the electroencephalographic patterns of patients who are in non-rapid eye movement sleep or under general anesthesia are very similar [22-24]. Their brain functional images are similar, too [22,25,26]. However, there are significant differences between normal sleep and general anesthesia. For example, it is easy to wake up a person from sleep. Consciousness recovery from general anesthesia can be achieved only after the anesthetics are eliminated from the brain. In supporting this system-based and sleep-like view of general anesthesia mechanisms, injection of the GABA_A _receptor agonist muscimol into the tuberomammillary nucleus, a brain region that is involved in sleep, but not the surrounding brain structures, causes hypnosis to rats [17]. This finding suggests that general anesthesia involves specific target proteins in specific brain regions.

## Volatile anesthetics-induced neuroprotection

### Neuroprotection induced by application of volatile anesthetics during brain ischemia

#### Animal studies

It was reported in 1963 that patients under cyclopropane anesthesia could tolerate longer temporary carotid occlusion without evidence of brain ischemia [27], suggesting that cyclopropane, a volatile anesthetic, may have neuroprotective effects. Numerous animal studies have been performed since then. Most of them show that volatile anesthetics are neuroprotective [3,4].

It was realized in 1990s that ischemic cell death is a dynamic process that can last for at least 2 weeks after brain ischemia in rodents [28]. Experts in the field recommend determining the long-term protective effects in pre-clinical studies [29]. To evaluate the long-term neuroprotective effects of volatile anesthetics when applied during brain ischemia, an early study subjected adult rats to middle cerebral arterial occlusion (MCAO) for 70 min in the presence or absence of 1.5 minimum alveolar concentrations (MAC) of isoflurane. One MAC is defined to be the alveolar concentration at which 50% subjects will not move in response to painful stimulation. Animals with isoflurane exposure during brain ischemia had smaller infarct volumes than those without isoflurane exposure at 2 days, but not at 14 days, after brain ischemia [30]. This result suggests that neuroprotective effects of isoflurane may not be long-lasting. However, a recent study showed that adult rats had smaller brain infarct volumes and better neurological outcomes evaluated at 14 or 28 days after brain ischemia if 1.8% isoflurane (~1.4 MACs) was applied during a 50-min or 80-min MCAO [31]. These findings strongly suggest that isoflurane provides long-lasting neuroprotection. One important difference between these two studies is that the early study permanently ligated the ipsilateral common carotid artery, which could cause chronic hypoperfusion to the brain regions including the previously ischemic brain tissues. The second study only temporarily occluded the common carotid artery during MCAO. This difference in creating MCAO may have resulted in the different conclusions regarding whether isoflurane-induced neuroprotection is long-lasting.

Multiple mechanisms have been proposed for the neuroprotection induced by volatile anesthetics [3,4]. Volatile anesthetics reduce metabolic rate of the brain. This effect should prolong the ischemic time that can be tolerated by the brain tissues and, therefore, should contribute to the anesthetics-induced neuroprotection. Volatile anesthetics reduce glutamate neurotoxicity due to their inhibition on glutamate receptors [32]. Since glutamate-induced over-excitation and the subsequent cell injury in the brain are a major mechanism for ischemic brain injury [33], anesthetics may provide neuroprotection through their inhibition of glutamate neurotoxicity. Also, increased intracellular calcium plays a critical role in ischemic brain injury [33]. Volatile anesthetics can regulate intracellular calcium concentrations [34], which may contribute to their neuroprotective effects.

#### Clinical studies

Michenfelder et al. [35] performed a retrospective data review of 2010 patients who had carotid endarterectomy with the monitoring of electroencephalography (EEG) and cerebral blood flow (CBF) from January 1, 1972 to December 31, 1985. Patients who were under isoflurane anesthesia had a lower critical CBF than patients under halothane anesthesia. The critical CBF was defined as flow below which the majority of patients had ipsilateral ischemic EEG changes within 3 min of carotid occlusion. Patients under isoflurane anesthesia also had a lower incidence of EEG evidence of brain ischemia than patients under halothane anesthesia. Since the critical CBF (20 ml/100 g brain tissue/min) of patients under halothane anesthesia is similar to that known to cause EEG changes of brain ischemia when no anesthetics are used (22 ml/100 g brain tissue/min) [35,36], these results suggest that isoflurane has neuroprotective effects in humans.

In a more recent study [37], 20 patients received desflurane as maintenance anesthetic during craniotomy. Patients were then randomized into two groups during cerebral arterial occlusion: 10 patients received thiopental and the other 10 received higher concentrations of desflurane to achieve EEG burst suppression. Patients in the desflurane group had better preservation of tissue oxygen saturation than patients in the thiopental group in the brain areas that were supplied by the occluded arteries. However, due to the small sample size, the study did not provide additional neurological outcome data.

In addition to these human results, two studies have used adult monkeys to determine volatile anesthetic neuroprotection [38,39]. These monkeys were subjected to a 5-h MCAO in one study and a 6-h MCAO in another study. However, there was no significant difference in infarct volumes and neurological functional outcome among the animals that were anesthetized by isoflurane, thiopental or nitrous oxide plus fentanyl. These anesthetics were used to achieve EEG burst suppression during brain ischemia.

Thus, there are very limited data suggesting the neuroprotective effects of volatile anesthetics applied during brain ischemia in humans. Up till now, there is no prospective and randomized clinical trial to determine these effects. The existing human and animal studies suffer from the issue that the effects of one anesthetic are compared to the effects of another drug or a group of drugs. Although it is difficult to overcome this difficulty in animal studies because animals are needed to be anesthetized during the creation of brain ischemia, this issue can be eliminated in human studies if surgical patients are not used in the study. However, multiple other issues in human studies can be difficult to control and confound the interpretation of the results. For example, there are differences in ischemic severity and brain regions among patients. Human studies also have more variables, such as baseline conditions and co-morbidities of patients. These issues should be considered when a prospective study is designed to determine the neuroprotective effects of volatile anesthetics.

### Neuroprotection induced by application of volatile anesthetics before brain ischemia

The concept "ischemic preconditioning" was introduced into the literature in 1986 [40]. Ischemic preconditioning describes a phenomenon in which episode(s) of short ischemia applied before a prolonged episode of ischemia reduce cell injury caused by the prolonged ischemia. This protection has two effective time phases. The acute phase starts minutes after the preconditioning stimulus and lasts for a few hours. The delayed phase starts hours after preconditioning stimulus and can last for many days [41]. These time windows relate when the prolonged episode of ischemia occurs for the protective effects to be present. Subsequently, various stimuli including volatile anesthetics have been found to induce a preconditioning effect in many organs [41,42].

We and other investigators have found that volatile anesthetics induce preconditioning effects in the brain (volatile anesthetic preconditioning-induced neuroprotection) [43-45]. We showed that the acute phase of this protection is anesthetic concentration-dependent. In case of isoflurane, this protection was maximized by the exposure to 2% isoflurane for 20 min [44]. Numerous studies have shown volatile anesthetic preconditioning-induced protection in the brain and spinal cord [46]. This protection appears to improve long-term neurological outcome in neonatal or adult rats [47,48]. Multiple molecules including signaling molecules, such as free radicals [49], intracellular Ca^++ ^[50], calcium/calmodulin-dependent protein kinase II [51], mitogen-activated protein kinase [43], protein kinase B/Akt [50], hypoxia-inducible factor-1α [52], and inducible nitric oxide synthase [45,53], have been implicated in the volatile anesthetic preconditioning-induced neuroprotection. Since volatile anesthetic preconditioning-induced neuroprotection has been reviewed previously [46], detailed description of this subject is not provided here. However, up till now, there is no clinical study on this subject, although an in vitro study using cell cultures has shown that isoflurane preconditioning also induces protection in human neuron-like cells [54].

### Neuroprotection induced by application of volatile anesthetics after brain ischemia

The phrase "ischemic postconditioning" was first used in the literature in 2003 [55]. It describes the protection induced by introducing short episodes of ischemia during the early phase of reperfusion after a prolonged episode of ischemia. Since this protection does not require the prediction on when the detrimental ischemia will occur, postconditioning-induced protection is considered to be more applicable in the clinical practice. In fact, post-treatment/postconditioning has been a common practice to provide medical care because most patients seek medical attention only after a disease/injury has occurred.

We have shown that application of isoflurane at the onset of reperfusion improves neurological outcome after a 90-min MCAO [56]. There are so far 6 published studies showing volatile anesthetic post-treatment-/postconditioning-induced neuroprotection [51,56-60]. This protection requires the application of volatile anesthetics within 1 h after the onset of reperfusion. Isoflurane, sevoflurane and desflurane at clinically relevant concentrations with the exposure times of 30-60 min have been shown to induce this protection. This protection can be induced in primary rat neuronal cultures, human neuron-like cell cultures, adult rats after transient focal brain ischemia, neonatal rats after brain hypoxia-ischemia injury or mice after an intracerebral hemorrhagic stroke. Various signaling molecules including glycogen synthase kinase 3β, Akt and mitochondrial K_ATP _channels have been implicated in this neuroprotection. However, there is no clinical study yet to determine whether volatile anesthetic can induce a postconditioning effect in the human CNS.

## Volatile anesthetics-induced neurotoxicity

### Animal studies

Studies on possible anesthetics-induced neurotoxicity can be divided into two groups based on their age interests: anesthetics-induced neurotoxicity in adult and neonatal animals.

Volatile anesthetics have been shown to affect the cognitive functions of adult animals. Various effects including improvement, impairment and no effects on cognitive functions have been reported in young adult animals [61-63]. Exposure of elderly rodents (18 months or older) to volatile anesthetics often impairs the cognitive functions of these animals [62,64]. Since β-amyloid peptide (Aβ) production and accumulation as well as neuroinflammation and neurodegeneration are considered to be underlying pathology for Alzheimer's disease (AD) [65,66], the most common form of dementia in elderly, the potential for volatile anesthetics to induce these changes has been investigated. Although volatile anesthetics are shown to increase inflammatory cytokines, Aβ and activated caspase 3 in the brains of young adult animals [61,67], no effects on cytokine production also have been reported in young adult mice [63]. However, up till now simultaneous determination of the AD-like brain changes and cognitive functions have been performed only in four studies. One study showed that halothane, but not isoflurane, increased amyloid plaque load in the brains of transgenic mice modeling for AD. Neither anesthetics increased active caspase 3 expression and affected the cognitive functions of these animals [68]. The second study showed that isoflurane anesthesia did not affect the inflammatory cytokine production in the brain and the cognitive functions of wild-type young adult mice [63]. Our study showed that exposure of young adult rats to 1.3% isoflurane for 2 h caused an acute and brief increase of interleukin 1β, an inflammatory cytokine, and active caspase 3 as well as a decreased neuronal density in the brain. This exposure also impaired the cognitive functions [61]. Similarly, we showed in another study that anesthesia with 1.3% isoflurane for 2 h impaired the cognitive functions of elderly rats. This impairment was abolished by lidocaine [69], a local anesthetic with anti-inflammatory property. Our studies appear to suggest that neuroinflammation and neurodegeneration play a role in the cognitive impairment. However, the causal relationship between these pathological changes and cognitive function impairments cannot be established yet based on the available data.

The potential detrimental effects of anesthetics on developing brains have become a hot topic in recent years due to various factors including the concerns on the safety of general anesthesia/anesthetics, the possible implications of these effects in children and their lives in future, and the investigators' efforts to increase the awareness of these effects in the communities. Although many studies have shown that volatile anesthetics can increase active caspase 3 expression and cell death in neonatal animals [5,70,71], a major concern on these in vivo studies is that many of these studies did not monitor the animals closely during the anesthetic exposure. This factor and the long exposure (≥ 6 h) often needed to see the effects raise the questions whether these in vivo studies faithfully simulate clinical situations and whether the neuropathology after the anesthetic exposure is indeed caused by anesthetics or changes occurred during anesthesia, such as hypoxia, hypocarbia and hypoglycemia. However, many in vitro studies also have shown that volatile anesthetics induce cell injury and death in cell cultures isolated from animals [72,73]. The environment of these cell cultures can be closely monitored and regulated. In addition, a recent study has shown that isoflurane induces cell apoptosis in the brain of neonatal rhesus macaque [74]. The anesthesia care of these macaques was similar to that of humans. Thus, the available evidence strongly suggests that a prolonged volatile anesthetic exposure (≥ 4 h) can cause cell injury and death in the neonatal brains of animals. However, it is not clear whether these findings from animals can be extrapolated to humans because volatile anesthetics at clinically relevant concentrations did not cause cell injury of human neuron-like cells or neuronal cell line in our study [75].

Associated with anesthetics-induced cell injury and death in neonatal animals, volatile anesthetics also have been shown to impair the cognitive functions of these animals [5,70,71]. Nevertheless, there is a lack of evidence for the causal relationship between the cell injury/death in the brain and the cognitive impairment in the animals after anesthetic exposure.

### Clinical studies

Clinical studies on the potential neurotoxicity of volatile anesthetics can be classified into three categories: the contribution of general anesthetic exposure to the development of postoperative cognitive decline (POCD) and AD in adult patients and learning and memory impairment in pediatric patients.

POCD is a fairly well-established clinical entity [76,77]. About 10% elderly patients have POCD at 3 months after non-cardiac surgery compared with ~4% patients with cognitive decline in the age-matched, non-surgery control group during the same period [76,77]. However, it appears that general anesthesia/anesthetics do not contribute significantly to POCD because patients under general anesthesia do not have an increased rate of POCD compared with patients under regional anesthesia [78,79]. On the other hand, a recent pilot study indicates that patients under isoflurane anesthesia may have a higher rate of POCD than patients under desflurane anesthesia after a lower extremity or abdominal surgery [80].

Many retrospective studies have been performed to determine whether anesthesia and surgery increase the risk for AD. Five of them including our study do not show any association of anesthesia and surgery with AD [81-85]. Two studies suggest a possible role of anesthesia and surgery in AD development. Among them, the first study found that patients who received anesthesia and surgery before the age of 50 years had an earlier onset of AD than those patients receiving anesthesia and surgery after they were more than 50 years old [86]. However, in a population-based case-control study, the investigators using the same group of AD patients showed that there was no difference in the rate of anesthesia and surgery between the AD patient group and the control group [82]. The second study showing a possible contribution of anesthesia and surgery to AD found that patients after coronary arterial bypass graft had an increased risk of being diagnosed with AD compared to patients after percutaneous transluminal coronary angioplasty [87]. Since coronary arterial bypass graft is a major surgery under general anesthesia and percutaneous transluminal coronary angioplasty is a procedure often under local anesthesia, these results suggest that general anesthesia and surgery may play a role in AD development. However, there was no difference in the percentage of patients who had coronary arterial bypass graft between AD patients and a control group in a population-based case control study [85]. Thus, evidence to suggest a role of general anesthesia and surgery in the AD development is very weak, if there is any. There is essentially no direct evidence on whether volatile anesthetics contribute to AD development.

A few retrospective studies have investigated the effects of general anesthesia and surgery occurred early in life (at the age of ≤ 4 years) on learning and memory functions of those children assessed later. No studies have shown that single exposure to general anesthesia and surgery causes learning and memory impairment in children. Two studies showed that exposure to multiple surgeries under general anesthesia increased the risk of learning disabilities [88,89]. The other 6 studies including two studies on twins did not show a link of general anesthesia and surgery to the development of learning disabilities [90-95]. The studies using twins are particularly powerful in this setting because they compared the learning abilities between the discordant twins (one twin was exposed and the other was not exposed to general anesthesia and surgery) [94,95]. This design eliminates many confounding factors, such as genetic background and social-economic status difference. Thus, the evidence to indicate a role of general anesthesia and surgery in the learning disabilities is weak. The effects of volatile anesthetics alone on the development of learning disabilities in children have not been determined yet.

## Prospective

Volatile anesthetics have been used in clinical practice for near 160 years and are still the most commonly used anesthetics worldwide. There are at least three very active research fields regarding volatile anesthetic effects in the CNS: anesthesia mechanisms, anesthetics-induced neuroprotection and neurotoxicity. The latter two effects seem contradictory to each other. Although there is solid evidence from numerous studies for volatile anesthetics-induced neuroprotection in animals, clinical data to support this effect are relatively weak. Data on volatile anesthetics-induced neurotoxicity in animal studies are accumulated rapidly. However, there are no human data for this effect yet. There is no prospective and randomized clinical trial yet to evaluate volatile anesthetics-induced neuroprotection or neurotoxicity. Such data may be very difficult or even impossible to get for anesthetics-induced neurotoxicity because it is not ethical to anesthetize a large number of people to determine anesthetic neurotoxicity. Although data from surgical patients may provide some hints on this effect, it is not possible to separate the anesthetic effects from the effects of many other factors associated with surgery. On the other hand, it is possible to design a clinical study to determine volatile anesthetics-induced neuroprotection by using non-surgical patients.

Logically, it is not difficult to understand that volatile anesthetics can have both neuroprotective and neurotoxic effects. It is often true that everything in our daily lives has two sides: the good and bad sides. For example, an appropriate amount of exercises is good for health. However, over-exercise is harmful. Volatile anesthetic preconditioning- and postconditioning-induced neuroprotection often requires anesthetic exposure for less than 1 h in animal studies [46]. This length of exposure has not been found to cause significant cell death. The shortest exposure for volatile anesthetics to cause brain cell injury is 2 h in both in vivo and in vitro studies. Most of them have anesthetic exposure for 4 h or longer [5,71].

Cell responses may be different when they are exposed to anesthetics in the presence or absence of insult/stress. Studies have shown that anesthesia/anesthetics reduce stress responses of surgical patients [96]. However, unnecessary inhibition of baseline cell activity by anesthetics, as is the case for almost all laboratory studies on the anesthetics-induced neurotoxicity, may cause imbalance of the cell activity, which ultimately leads to cell injury. Since virtually almost all currently used anesthetics have been shown to cause brain cell injury in the laboratory studies [5,71], one has to wonder what anesthesiologists can use to anesthetize the patients and how reliable those laboratory studies are to simulate clinical situations. In addition, clinicians have to weigh the risk and benefit of using general anesthetics for surgical patients. Clearly, general anesthesia is necessary and beneficial in most of these cases.

## Conclusions

Volatile anesthetics are commonly used drugs. The mechanisms for them to induce anesthesia are not fully understood. The most popular theory in the field now is the protein hypothesis: anesthetics work on specific protein targets to induce anesthesia. Currently, volatile anesthetics-induced neuroprotection and neurotoxicity are two hot research topics. These two effects seem contradictory to each other and each is supported by sufficient laboratory studies. However, clinical data to support the neuroprotective effects are relatively weak. There are no clinical data yet to suggest volatile anesthetics-induced neurotoxicity.

## Abbreviations

Aβ: β-amyloid peptide; AD: Alzheimer's disease; CBF: Cerebral blood flow; CNS: Central nervous system; EEG: Electroencephalography; GABA: γ-aminobutyric acid; MAC: Minimum alveolar concentrations; MCAO: Middle cerebral arterial occlusion; NMDA: N-methyl-D-aspartic acid; POCD: Postoperative cognitive decline.

## Competing interests

The author declares that they have no competing interests.

## Authors' contributions

This manuscript was solely prepared by ZZ, MD, Ph.D.
